# Integrating community-based verbal autopsy into civil registration and vital statistics (CRVS): system-level considerations

**DOI:** 10.1080/16549716.2017.1272882

**Published:** 2017-01-31

**Authors:** Don de Savigny, Ian Riley, Daniel Chandramohan, Frank Odhiambo, Erin Nichols, Sam Notzon, Carla AbouZahr, Raj Mitra, Daniel Cobos Muñoz, Sonja Firth, Nicolas Maire, Osman Sankoh, Gay Bronson, Philip Setel, Peter Byass, Robert Jakob, Ties Boerma, Alan D. Lopez

**Affiliations:** ^a^ Department of Epidemiology and Public Health, Swiss Tropical and Public Health Institute, Basel, Switzerland; ^b^ University of Basel, Basel, Switzerland; ^c^ Melbourne School of Population and Global Health, University of Melbourne, Carlton, Australia; ^d^ Department of Disease Control, London School of Hygiene and Tropical Medicine, London, UK; ^e^ African Field Epidemiology Network (AFENET), Kisumu, Kenya; ^f^ National Centre for Health Statistics, Centres for Disease Control and Prevention, Hyattsville, MD, USA; ^g^ CAZ Consulting, Geneva, Switzerland; ^h^ Africa Centre for Statistics, United Nations Economic Commission for Africa, Addis Ababa, Ethiopia; ^i^ INDEPTH Network, Accra, Ghana; ^j^ School of Public Health, University of Witwatersrand, Johannesburg, South Africa; ^k^ Vital Strategies, New York, NY, USA; ^l^ WHO Collaborating Centre for Verbal Autopsy, Umeå Centre for Global Health Research, Epidemiology and Global Health, Department of Public Health and Clinical Medicine, Umeå University, Umeå, Sweden; ^m^ MRC-Wits Rural Public Health and Health Transitions Unit (Agincourt), School of Public Health, University of Witwatersrand, Johannesburg, South Africa; ^n^ Department of Health Statistics and Information Systems, World Health Organization, Geneva, Switzerland

**Keywords:** Mortality surveillance, cause of death, systems integration, information technology, health information systems, process mapping, international classification of disease, Sustainable Development Goals

## Abstract

**Background**: Reliable and representative cause of death (COD) statistics are essential to inform public health policy, respond to emerging health needs, and document progress towards Sustainable Development Goals. However, less than one-third of deaths worldwide are assigned a cause. Civil registration and vital statistics (CRVS) systems in low- and lower-middle-income countries are failing to provide timely, complete and accurate vital statistics, and it will still be some time before they can provide physician-certified COD for every death.

**Proposals**: Verbal autopsy (VA) is a method to ascertain the probable COD and, although imperfect, it is the best alternative in the absence of medical certification. There is extensive experience with VA in research settings but only a few examples of its use on a large scale. Data collection using electronic questionnaires on mobile devices and computer algorithms to analyse responses and estimate probable COD have increased the potential for VA to be routinely applied in CRVS systems. However, a number of CRVS and health system integration issues should be considered in planning, piloting and implementing a system-wide intervention such as VA. These include addressing the multiplicity of stakeholders and sub-systems involved, integration with existing CRVS work processes and information flows, linking VA results to civil registration records, information technology requirements and data quality assurance.

**Conclusions**: Integrating VA within CRVS systems is not simply a technical undertaking. It will have profound system-wide effects that should be carefully considered when planning for an effective implementation. This paper identifies and discusses the major system-level issues and emerging practices, provides a planning checklist of system-level considerations and proposes an overview for how VA can be integrated into routine CRVS systems.

## Background

Real-time and accurate statistics on mortality and COD are essential for the development of national health and population policies, and underpin the ability of countries to respond to emerging health threats and epidemics. The United Nations (UN) and World Health Organization (WHO) standards for mortality statistics require the recording in the civil registry of all deaths, by age, gender, date and place of occurrence, along with medical certification of the cause of death (COD) by trained physicians according to the International Classification of Diseases (ICD). The information should be regularly compiled into vital statistics through national civil registration and vital statistics (CRVS) systems. Although there has been growing momentum to strengthen CRVS systems in recent years [1–4], it will still be some time before the routine CRVS systems currently present in low- and lower-middle-income countries will be able to ensure physician-certified COD for a significant proportion of deaths. This is especially so for those that occur in rural or remote areas, owing to the lack of trained physicians and the fact that at least two-thirds – and often more than 80% – of deaths occur at home. Summary COD statistics available in such countries are often biased since they derive mainly from deaths in health facilities and in urban settings, which are not representative of the experience of the general population [5]. As a result, CRVS systems are unable to generate data that are sufficiently reliable and representative for public health policy and planning purposes [6]. This is of particular concern given the dynamics of the demographic and health transitions occurring around the world and the need to document progress towards the Sustainable Development Goals [7–9].

Drawing on the UN Sustainable Development Goals, the WHO targets for universal civil registration of births and deaths, including COD, the World Bank CRVS targets [10], the UN Economic and Social Commission for Asia and the Pacific [11] and the UN Economic Commission for Africa’s regional programmes supported by ministerial level engagement [12], there are new goals to achieve significant improvements in CRVS reporting by 2020, such that:

- 60% of deaths in a given year are continuously notified, registered and certified with key characteristics
- 80% of deaths in hospitals have COD reliably determined and officially certified in real time
- 50% of deaths in communities have probable COD determined in real time, and collection systems designed in a representative way.

To address the last goal, over the past decade there has been significant progress in the development of verbal autopsy (VA) methods to identify the probable COD in the absence of a physician. VA involves an interview – after a culturally acceptable mourning period – with an appropriate next of kin, family member or care-giver of a deceased person to collect information on signs and symptoms experienced by the deceased prior to death. This information is then analysed, either by a physician or, more innovatively, by automated computer algorithms, to yield a probable COD that can be coded according to ICD standards [5,13–20].

There is extensive experience in the use of VA in research settings for determining the COD [21–23]. This experience has led to advances in the VA questionnaire design [19,24,25], in data capture on mobile devices [19,26], and in the use of computer algorithms for determining and coding the probable COD [13,20,27–30]. These advances make paper-based and mobile tablet-based VA at community level an increasingly available and effective substitute for physician-certified COD in settings where a significant proportion of deaths occur outside hospitals. The methodological developments have been accompanied by efforts to significantly reduce the time and cost of VA interviews and ascertainment of COD, thus greatly increasing the potential of automated VA methods to be applied in routine CRVS systems to cost-effectively monitor, in real time, COD patterns in populations [14,19,31,32].

VA is an imperfect tool for ascertaining COD, but it is the only alternative in the absence of medical certification [29,33–37]. For this reason, and in light of the technological advances that make scaled application feasible, there is growing interest in applying paper-based or mobile tablet-based VA beyond the research setting, as a routine component of CRVS operations where physician-certified COD reporting is currently rare or absent [38]. Community VA may also prove useful in hospital settings to establish COD for cases that are ‘dead on arrival’, or shortly thereafter, where post-mortem pathological examinations are unavailable, or that are certified to non-specific causes such as ‘cardiorespiratory failure’ that are of little value for public health decision making [39].

Despite many important technical advances, the introduction and sustainable integration of VA into CRVS systems is more than just a technical challenge. A number of systems integration and methodological issues will need to be carefully addressed during the planning, piloting and implementation stages of any such endeavour. VA is nascent in its use within routine CRVS settings. This paper describes emerging practices from which we can begin to establish an evidence base for best practices and recommendations. The goal of the paper is to describe how VA processes can be integrated into a CRVS system; as such, it is also important to highlight implications of VA on the civil registration system. In this paper, we identify and discuss some of the more important system-wide issues. These fall broadly under the domains of governance, design, operations, human resources, financing, infrastructure, logistics, information technologies and data quality assurance.

## Proposals

### Governance issues

**Figure 1.**
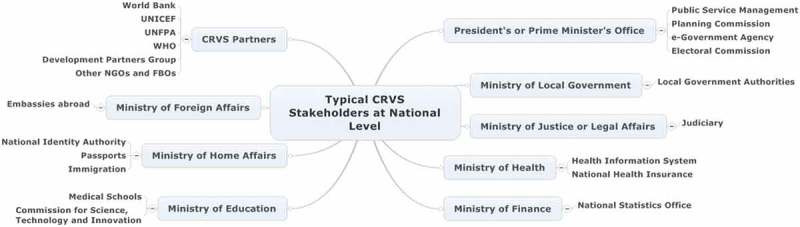
Typical civil registration and vital statistics (CRVS) stakeholders at national level. NGOs = non-governmental organizations; FBOs = faith-based organizations.

Owing to the multiplicity of actors and sub-systems involved in the production of complete and reliable vital statistics and the importance of civil registration for policy making, CRVS systems engage an extraordinary range of critical stakeholders across multiple sectors. See Figure 1 for a typical example. Such complexity presents numerous governance challenges.

#### National CRVS Policy and Coordinating Committee

A high-level National CRVS Policy and Coordinating Committee often best governs effective CRVS systems, because of the many sectors, government agencies and partners concerned [40]. These typically include the ministries of local government, health, justice and the interior, as well as specialized agencies or bureaux such as statistics, national identity (ID), e-government, national health insurance, and others concerned with various population registries (e.g. electoral commissions). The chair of this high-level committee is a critical choice, and often reports directly to the highest level of government above-the-line ministries, such as a planning commission in either the president’s or prime minister’s office, or is the minister responsible for civil registration. This will enable the committee to have convening authority for those in other ministries and agencies. Where there is a national ID system, with or without biometric identifiers, it is beneficial that it is also represented on the high-level committee. In the absence of such cross-sectoral involvement, there are risks that parallel data capture systems and conflicting vital statistics will occur, leading to inefficiencies and (potentially) undermining demand for civil registration services. The National CRVS Policy and Coordinating Committee could typically be concerned with:

1. the coordination and development of the CRVS system, including establishing its long-term vision and funding strategy
2. overseeing efforts to define and harmonize stakeholder functions, systems and data sources, ensuring standards for interoperability of information flow and databases
3. keeping political, legal and regulatory environments supportive of the CRVS system throughout its evolution, in particular as new technologies emerge
4. monitoring opportunities and new developments to improve the efficiency of the CRVS system, such as decisions for incorporating VA as an integral part of CRVS
5. overseeing any interagency technical working group(s) or sub-committees on CRVS that undertake day-to-day work of implementing CRVS programmes and plans.

#### National sub-committee on mortality and cause of death

Given the importance of accurate, real-time mortality data for public decision making, the National CRVS Coordinating Committee could convene a National Sub-committee on Mortality and Cause of Death, responsible for developing and overseeing a strategy for ensuring that nationally representative mortality data are available to, and used by, decision makers for planning. Such a sub-committee could be under the auspices of the Ministry of Health. Among other tasks, this mortality Sub-committee or equivalent would advocate for the need to monitor COD and develop materials and plans to support this objective for both deaths in health facilities and deaths outside health facilities, including deaths due to unnatural causes. Advocacy may entail explaining the need to stay abreast of epidemiological change and transition and to measure the mortality-specific indicators that could be monitored in response to Sustainable Development Goals and targets for health [7]. The Sub-committee would also advocate for the importance of real-time tracking of mortality in order to continuously update national population registers, ID databases and electoral rolls.

The Sub-committee would probably establish a Task Force on CRVS-VA or similar mechanism to address the particular challenges involved in generating information on COD using VA. Typically, the CRVS-VA Task Force would deal with the legal, design, operational, technical and systems issues that could be addressed in preparing plans for CRVS VA integration, including:

1. commissioning the detailed process mapping of how death and COD are captured at health facilities and outside health facilities, and using these to conceptualize how VA could be integrated
2. addressing the legal, regulatory and ethical issues that are likely to arise in relation to non-physician ascertainment of COD
3. making recommendations to the National CRVS Planning and Coordinating Committee via the Mortality Sub-Committee regarding the legal framework that is likely to be most appropriate to support VA implementation in the country
4. guiding the detailed design and piloting of integrated VA in CRVS, considering the financing, human resources, logistics, quality control and information technology (IT) needs, including resolving technical issues, e.g. choice of questionnaire, algorithms and sampling strategy
5. developing recommendations to the National CRVS Planning and Coordinating Committee via the Mortality Sub-committee for the plan and budget for integrating VA into the CRVS system
6. monitoring and evaluating the implementation and scale-up of VA in CRVS
7. addressing the question of how VA-ascertained COD data from communities will be analysed along with physician-certified hospital deaths to permit the CRVS to provide the most comprehensive picture of CODs in the country.

#### Business case

Countries may also want to commission the preparation of a business case to justify using VA as a method to ascertain the most likely underlying COD in selected populations. Such a business case may point out the substantial emerging advantages of digital data collection for automated VA on mobile tablet devices instead of relying on paper for interviews at household level. Electronic data capture at household level: (1) vastly facilitates the work of the VA interviewer owing to automated skip logic navigation through the complex questionnaire; (2) reduces error rates in data collection owing to control of valid data entry for respective variables; (3) reduces the cost of manual double data entry from paper; and (4) eliminates transcription errors when transcribing data from paper to computer databases. There are also significant savings in both time and cost by taking advantage of computer coding in place of physician coding of the VA results to determine the underlying COD [15,41,42]. The business case will need to consider higher level benefits from integrating VA in CRVS, such as strengthening community death notification as part of CRVS, increasing the completeness of both fact of death and COD data in CRVS, and the use of community-based mortality statistics to better target programme implementation and funds.

### Design and operational issues

#### Integrating processes

Emergent experience is showing that preparing detailed process maps is a valuable early step in designing the integration of VA into CRVS systems.^1^ ^1^Unpublished observations from the Bloomberg Data for Health Initiative in 15 countries. These maps depict all major steps, processes and activities related to notifying, registering and certifying deaths in the CRVS system. Process mapping describes the major actors or agencies involved, from the family through the local authorities, local health system, civil registry system, eventually to national databases and vital statistics. The process maps also help to establish efficient business functions, reporting forms, standard operating procedures, requirements, rules and information flows concerned with capturing the mortality event. These ‘blueprints’ of the CRVS architecture allow all stakeholders to have a common understanding of the current system ‘as-is’. These maps are helpful to enable effective participation in designing how the CRVS and VA steps (declaration, notification, registration, certification, VA interview, VA analysis, linking COD and fact of death, validation, quality control, data transfer, production and dissemination of vital statistics and reports, etc.) are to be integrated into the CRVS system. In addition, process maps can be useful in supporting the digitization of CRVS (http://www.crvs-dgb.org/en/) and in ensuring the interoperability of data systems, for example, when integrating VA information flows into both the CRVS system and digital health information systems such as DHIS2 (https://www.dhis2.org/). The process maps facilitate the identification of pathways to enable the transition from ‘as-is’ CRVS processes design to ‘as-desired’ processes that can accommodate the addition of routine VA and are key to undertaking a meaningful legal review, which is an important step in planning the addition of VA to CRVS.

#### Sampling

Ideally, VA could be conducted to estimate the COD in all out-of-facility deaths where there is no physician certification. Costs savings can be achieved by conducting VA on a representative sample of all deaths or in a representative selection of registration administrative areas [43–45]. Some countries with large populations conduct a VA only on samples of deaths in communities by Sample Registration Systems (SRS) or Sample Vital Registration with Verbal Autopsy (SAVVY) to determine statistically useful cause-specific mortality fractions at population level (e.g. China, India, Tanzania, Indonesia) or in health facilities to improve the quality of data from health facility deaths (e.g. Brazil, Thailand). At present, the SRS and SAVVY sampling units (e.g. India, Tanzania) are census enumeration areas. However, for VA to be better integrated into CRVS, the sampling units could be the registration administrative units. When sampling is done, sample size and designs need to be considered carefully to avoid political concerns, ensure representativeness, and provide affordable and cost-effective scenarios. Other challenges faced by sample VA approaches include ensuring that the deaths and associated CODs are well linked in the CRVS, and that the sampling scheme does not limit provision of sub-national data on COD, which is important in countries that have large sub-national differences.

#### Community operational considerations

**Figure 2.**
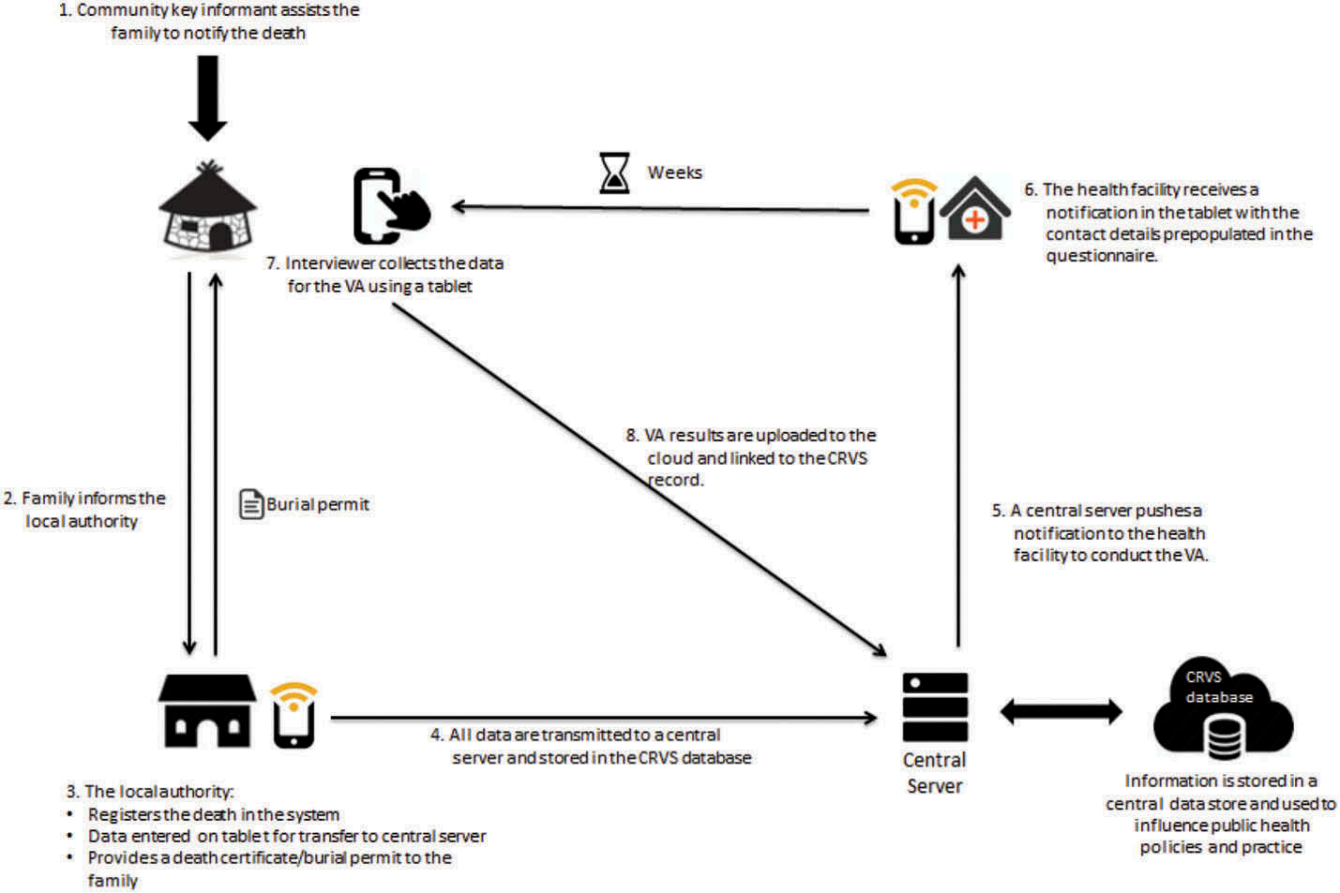
Potential verbal autopsy (VA) processes in a civil registration and vital statistics (CRVS) system.

**Figure 3.**
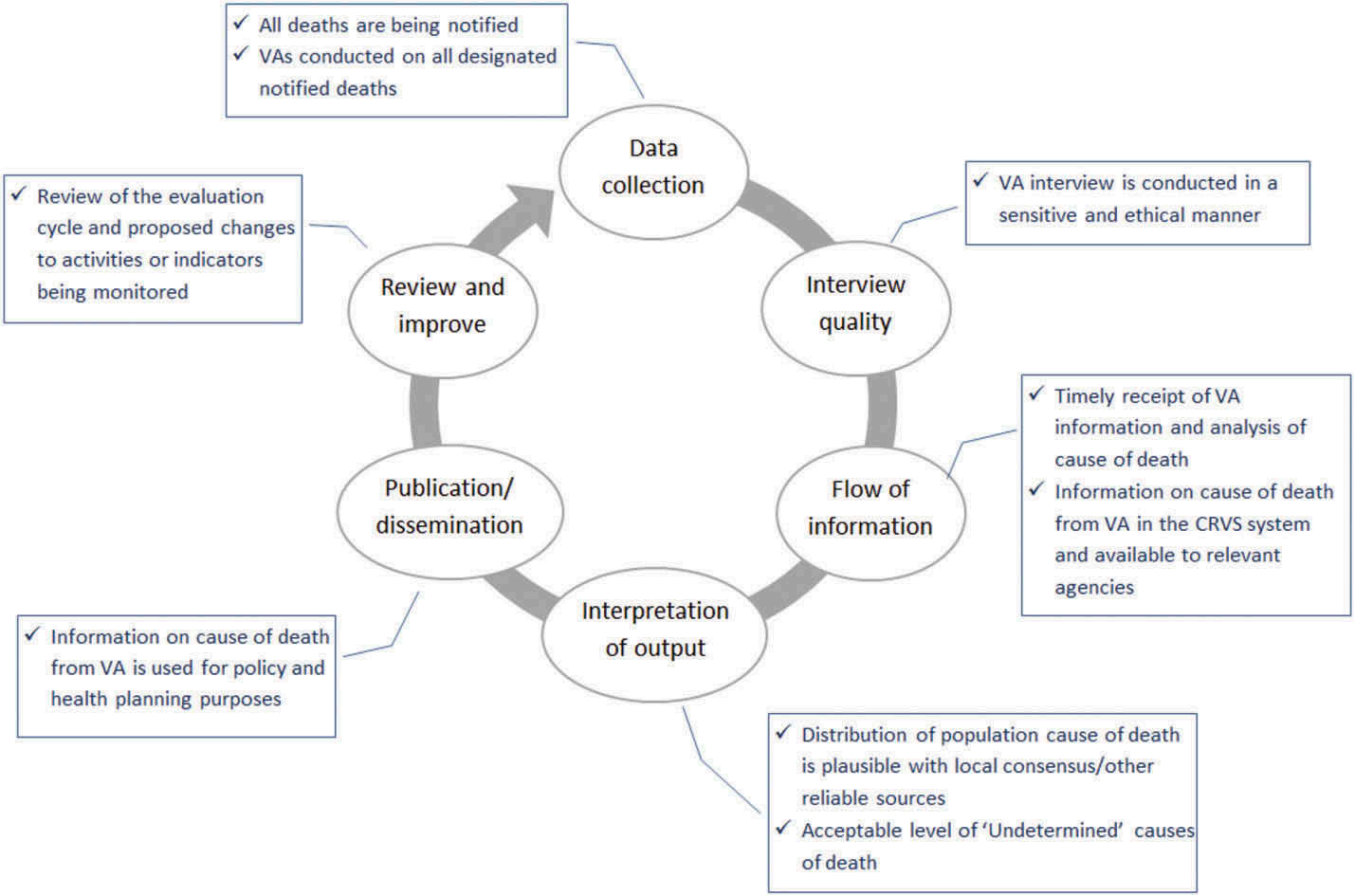
Example of a monitoring and evaluation cycle for verbal autopsy in civil registration and vital statistics (CRVS). VA = verbal autopsy.

**Figure 4.**
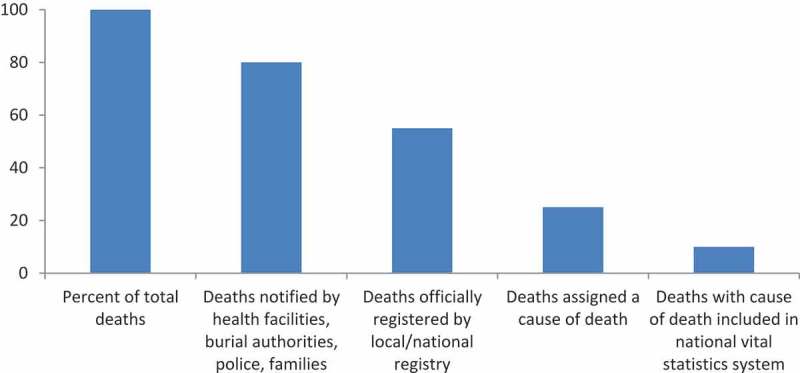
Potential data loss from notification of deaths to production of mortality statistics. Hypothetical scenario common to low-income countries.

**Figure 5.**
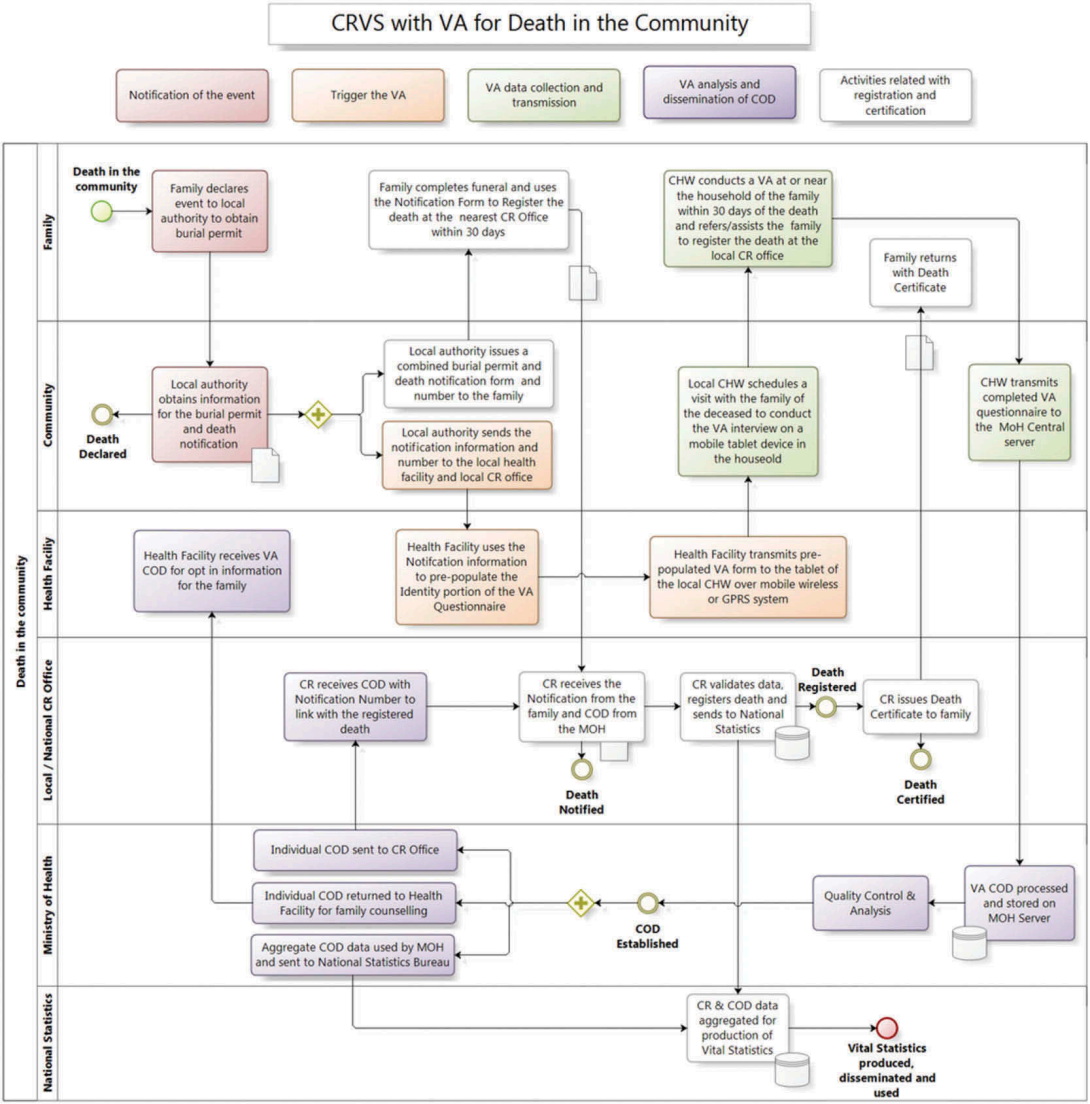
Generic processes for integrating community verbal autopsy in a hypothetical civil registration and vital statistics (CRVS) setting for community deaths. VA = verbal autopsy; COD = cause of death; CR = civil registry; CHW = community health worker; MoH = Ministry of Health; GPRS = General Packet Radio Service.

The following is a brief overview of how VA could be integrated as part of a typical CRVS system for deaths in the community (Figures 2–5). One key principle proposed here is that the notification of the fact of death to the civil registration system could occur before the VA is scheduled and conducted. The VA could then be conducted on a death that already has been assigned an official notification, registry number or unique ID. This reduces potential problems in later data linkage and the risk of double counting. It also enables data linkage to be conducted to estimate possible bias and gaps in notification and registration. The processes for declaring or notifying deaths, particularly those in the community, become the starting point for the new VA processes. This is likely to require reconsideration of how vital event notification is conducted at community level. This step is currently a passive step in most CRVS systems and it will need to become an active step.

The critical challenges for the design and operationalization of VA in CRVS are therefore: (1) the immediate identification of death events as they occur in the population (usually in disadvantaged, remote rural communities); and (2) ensuring that the death, with its associated fact of death details [ID (where relevant), name, age, gender, address, location, date of death, etc.] are already registered in the CRVS system before the VA is conducted (usually not before 30 days or later than 3 months from the date of death, although up to 12 months may still be acceptable) [46].

#### Notification

Most CRVS systems are passive, relying on families to come forward to register vital events and placing the onus on the family to report events, which results in low notification and registration rates. Notifications of deaths to the CRVS system can be increased through a variety of active case-finding methods, including village authorities or chiefs, community key informants, village volunteers, community outreach workers, or surveys or surveillance systems. Such methods are, however, likely to be costly and difficult to sustain in a routine system. In some countries, short message services (SMS) on mobile phones to notify events from the community to the registration authority or health facilities have been tried [47,48]. Hybrid approaches that use a combination of reporting through community-based entities such as health facilities, burial authorities or local chiefs, along with verification and official registration by family members, may be more cost-effective. Implementing such a model will be likely to require changes to the existing CRVS system. Where reporting of all deaths is required and enforced, however, these notifications can also be used to trigger scheduling of the VA and official registration of the death. Events could also be notified by proxy procedures such as reviewing the issuance of burial permits by local authorities where this has high coverage and enforcement. The VA questionnaire, either on paper or on a mobile tablet computing device, can then be pre-populated with the minimum necessary notified fact of death information, such as name, age, gender, date of death, location, and notification serial number or civil register or personal ID number for the deceased, before the trained VA interviewer is dispatched to the deceased’s household.

#### VA interview

The paper- or mobile tablet-based VA questionnaire is usually conducted in a place, at or near the household of the deceased, where those family members most familiar with the circumstances of the death are available and comfortable to respond freely with proper consideration to locally appropriate protection of confidentiality. The interview responses are best captured digitally in a mobile tablet computer to ensure quality, increase the efficiency of data collection and reduce the cost of data entry. The interviewer should not be tasked with interpreting, providing or counselling on the COD. The family could be referred to the appropriate local health facility where they can be provided at a later date with an interpreted COD from the VA on an opt-in basis, at least to broad-cause level, with counselling. However, the VA interviewer will need to be well trained in an approach to managing the interview process that is ethically and culturally appropriate, including familiarity with principles of grief counselling for self-care and for managing the emotionally sensitive interview process [49,50]. At the end of the interview, the contents of the completed VA interview questionnaire should be automatically encrypted and transmitted securely from the VA tablet to a dedicated server at central level or otherwise linked to the CRVS database.

#### VA interpretation

External to the mobile tablet-based VA questionnaire at the data server level, the probable underlying COD (and where feasible the associated ICD-10 code) can be imputed through an appropriate validated algorithm. This is likely to be the most feasible method for routinely estimating population COD structure, which can be more accurately specified than an individual diagnosis owing to compensating diagnostic errors standards [5,13–20].

To comply with legal requirements in certifying the individual COD, physician certification may still be required in some countries for CODs that are added to CRVS civil registers [33]. In such scenarios, a physician could be asked to read and consider the VA interview results and sign off, or modify, the computer-generated cause. Even where a physician is not required by law to certify the COD, the potential implications of automated VA results applied to individual records in the CRVS, for example with regard to insurance claims or inheritance, could be considered. Gouda et al. discuss these and other ethical issues around VA [51]. Thus, legal and regulatory review may be required as an early step in considering VA integration into CRVS. The cause, with its ICD-10 code, could then be linked and added to the CRVS civil record according to the registry number and transmitted to both the vital statistics authority and the Ministry of Health as required. A check box with other detail could be added to the record to indicate that the cause was derived by a VA algorithm that is clearly specified by name and version. See Figure 2 for an overview of the approach, and Figure 5 for a more detailed generic flow diagram of VA integration into CRVS.

### Human resource issues

It is important to have a profile of the existing CRVS human resources and needs. Introducing community VA will require new functions to be added to existing human resource cadres or new cadres to be recruited, trained, supplied, supervised and possibly remunerated. Those at the front line who identify deaths in the community can be community volunteers (key informants) who would not need financial compensation, or paid community outreach workers whose job descriptions can be expanded to include notification of births and deaths. Including births in the notification process is important since high-mortality rural populations have relatively high neonatal mortality rates that are often underestimated because pregnancies and births go unrecorded and unregistered.

Job descriptions, training plans and training materials need to be developed for the new functions or cadres. It is expected that the addition of mobile community-based VA will greatly increase the number of deaths, and CODs, that enter the CRVS system, and this will increase the workload of the existing CRVS system at higher levels. This consideration needs to be estimated and planned for to ensure that the increased workload can be absorbed and managed.

VA interviewers can be selected from local community members who have secondary school qualifications and could be working in some official capacity within the country’s government, as they are facilitating an official function of the country’s CRVS system. Owing to the relatively infrequent nature of mortality in geographic areas accessible to individual community interviewers, VA interview work is not generally a full-time occupation, and these tasks can be added to position descriptions of other community outreach workers who may be employed by local authorities. While some health expertise background may be helpful for VA interviewers, it is not necessary that highly trained health-care providers (e.g. physicians, clinical/medical officers or nurses) serve as VA interviewers, as trained lay interviewers perform this work adequately in research settings [52].

Countries that already host research systems conducting VA (e.g. Health and Demographic Surveillance Systems) will have an advantage by being able to tap into these skills to help develop the human resource plan, assist in training and quality control [53], and participate in respective committees (see ‘Governance issues’, above).

In Table 1 we outline an approximation of the human resource cadres and numbers required. This assumes that a country has a national crude death rate of 6–7 per 1000, but that the community VA would likely to be conducted for rural deaths in more disadvantaged populations where the crude death rate may be 9 or more per 1000. It assumes that VA interviewers will be community based and that the frequency of deaths in their catchment area will be relatively low (approximately one death per week per interviewer), so that their work will not be full time. An alternative would be to have fewer VA interviewers with larger catchment areas. This would need to be traded off against the higher costs and greater difficulty for such workers to find and reach households, and the need to provide them with transport. There is the additional trade-off of having a sufficient frequency of VA work to maintain their skills in conducting VA, while not overloading and crowding out their other functions.

**Table 1.** Suggestions for human resource cadres needed to operate verbal autopsy (VA) in a civil registration and vital statistics (CRVS) system.

Cadre	Number/1,000,000 population (for 9000 deaths per year)	Level
Community key informants for notification	1000	Unpaid volunteer, part time
or community outreach or health workers	1000	Paid by government, part time
VA interviewers	250	Paid, part time or per event
VA regional supervisors	25	Paid, part time
VA physician coders or signers – optional	6	Paid per event, part time
VA IT, logistics and help desk	2 per country	Paid, full time
VA analyst	1 per country	Paid, full time
VA national coordinator	1 per country	Paid, part time

### Financing issues

Because there is limited experience in integrating community VA into CRVS, it is difficult to estimate the costs of routine CRVS VA. In sample registration systems with VA, such as in India [54], Indonesia [55], Tanzania [43] and Ethiopia [56], costs per VA conducted are estimated between 5 and 15 USD per case. In some cultures, condolence fees or gifts may be expected when visiting households after a death, and this may need to be considered. Formal costing studies in these CRVS-VA pioneering countries will be valuable, as other countries will want to know the financial and economic costs of adding VA to their CRVS system. As countries gather more experience at scale, more costing studies are required and a CRVS costing tool needs to be developed to guide country decision making. Countries will want to know the total system cost versus the added value of information gained. We estimate that the main cost drivers will be training and retraining, IT infrastructure, remuneration of VA interviewers and supervising VA interviewers.

### Infrastructure and logistics issues

The existing infrastructure and its gaps should be well known and mapped. The process mapping of CRVS alone, and CRVS plus VA, developed at the design stage is valuable for understanding these gaps in infrastructure, logistics and IT. This is particularly so when adding new cadres to operate the VA system, since they will have office and infrastructure needs additional to the current CRVS infrastructure.

National Identification agencies in low-income countries are currently building physical and IT infrastructure. CRVS agencies need to be cognizant of these developments and seek synergies, particularly for the planned introduction of VA into CRVS systems.

Infrastructure for CRVS and VA in particular will increasingly depend on mobile data and wireless network capacity. These also need to be mapped in order to forecast what will be needed to support current and developing infrastructure.

### Data and information technology systems

Increasingly, VA for CRVS will be a digital rather than paper-based undertaking from the point of data capture using tablet or smart-phone devices. VA questionnaire instruments need to be adapted and translated in a way that captures the correct medical concepts in locally understood terminology, yet does not compromise the instrument’s established performance. Detailed guidance for interviewers and translators is currently being developed by the WHO [57]. Cognitive testing to ensure that respondents understand the questions as they are intended is also recommended [58].

Some of the instrumentation challenges include the fact that tablet interview script language and the language in which the interviewer interacts with respondents may not be the same, or the fact that some countries use different calendars.

Devices will need to link to servers, either locally or centrally, and the flow of data needs to be designed and managed from an enterprise architecture (EA) perspective. EA is the industry standard for designing interoperability in information and communication applications. The UN Economic Commission for Africa has developed guidance specifically for how to employ EA for CRVS digitization [59]. Open-source software such as Open Data Kit (ODK) [60] is ideally suited to developing the necessary software solutions for data collection and data transmission. However, e-governance, data security, confidentiality and data encryption issues should be addressed. Some countries may want to feed results into or via their digital health information infrastructure, such as DHIS2 [61]. The IT systems could have dedicated customized dashboards with controlled level access for various managers and implementers. The IT arrangements and specifications for such servers and communications are available [62]. Aggregate results will need to be managed in a timely fashion. Skills for these functions are not often available, hence the inclusion of dedicated IT and logisticians in human resources. In locations where mobile and wireless networks are not sufficiently reliable, an off-line method, involving the VA interviewer bringing their tablet to a health centre and downloading the VA manually through a USB connection to a computer, is a less IT-intensive solution.

#### Access to data

There are important legal and ethical dimensions related to COD data in CRVS systems [51]. VA will be an additional source of such data and should be considered sensitive, personal medical data, accessible by the smallest necessary number of agencies and their agents for clearly defined purposes. For this reason, a legal and regulatory review (See Table 2) is an important early step in planning and designing the VA integration. As with medically certified CODs from health facilities being captured in CRVS systems, this review could specify levels of access to VA COD data on a need-to-know basis, including the need for encryption of data during transmission to the servers, and server security and access protocols. To protect confidentiality, data can be anonymized, minimizing access to personal identifying information and linking the COD via unique identifiers only at levels that need to know. Population-level estimation of COD does not require personal identifying information, which can be stripped before COD data are made available to those agencies conducting population-level analyses. The algorithms that estimate the VA COD are not resident on the VA interviewer’s tablet computer and so the cause cannot be known or communicated to the family at that point. But in some instances, the health system may be obligated to inform next of kin of the likely COD from the VA on an opt-in basis to satisfy family demands. If so, provisions should be made for ensuring secure transmission of the identity-linked result from the VA analytic server back to the appropriate health-facility/health-worker level, where confidential counselling can be provided. Although challenging, it is important to make provision to maximize the chances that the information is managed securely.

**Table 2.** Planning checklist of system-level considerations for planning verbal autopsy (VA) integration in civil registration and vital statistics (CRVS) systems.

Ↄ Ensure that a high-level National CRVS Policy and Coordination Committee is in operation;
Ↄ Ensure that the relevant authorities, agencies or ministries for civil registration, statistics, local government and health and are engaged collectively for CRVS;
Ↄ Ensure that a Comprehensive CRVS Assessment has been conducted in the past 4 years and has been used to develop a national CRVS vision and strategy or is being planned;
Ↄ Set up a National Sub-committee on Mortality and Cause of Death;
Ↄ Establish a task force for VA implementation reporting to the National Sub-committee on Mortality and Cause of Death;
Ↄ Ensure that detailed process mapping of CRVS processes for registration of death in health facilities and death in communities has been done as part of the comprehensive assessment, and if not, prepare such process maps;
Ↄ With all relevant stakeholders, use these process maps of notification and registration processes of death in the community as a base to develop the plan of implementation for how VA would be integrated into a modified set of processes;
Ↄ Prepare an investment case to justify using VA as a method to increase notification and registration of deaths and ascertain underlying cause of death;
Ↄ Consider a legal and regulatory review of the implications of VA in CRVS as an early step in the plan;
Ↄ Apply the enterprise architecture Digital CRVS Guidebook to assess the additional IT needs (http://www.crvs-dgb.org/en/);
Ↄ Map the existing CRVS and DHIS2 IT infrastructure and its gaps;
Ↄ Seek synergies with existing IT for population registration efforts (i.e. National Identification agencies);
Ↄ Determine how mobile tablets will be supported, maintained and securely transmit/receive data (wireless, General Packet Radio Service, etc.);
Ↄ Design data flow and quality assurance mechanisms;
Ↄ Ensure that e-governance, interoperability, data security, confidentiality and data encryption issues addressed;
Ↄ Decide how VA-coded deaths will be distinguished from medically certified deaths in aggregate databases;
Ↄ Decide on scale (sample system or full coverage) and phased introduction;
Ↄ Use a VA costing tool to develop the start-up and annualized budgets;
Ↄ Prepare a profile of the existing CRVS human resources and needs;
Ↄ Develop job descriptions, training plans and training materials for new and revised positions;
Ↄ Plan for an increase in the workload for existing staff;
Ↄ Consider adding VA functions to existing position descriptions of community workers;
Ↄ Develop a training programme for Master Trainers, Trainer of Trainers, and training of VA supervisors, interviewers and analysts;
Ↄ Prepare a monitoring and evaluation plan for the new VA processes, including the use of VA costing tools to document costs and an independent quality assurance mechanism;
Ↄ Work with stakeholders to develop a learning platform for phased introduction and assemble necessary funding.

### Quality assurance

#### Monitoring and evaluation

Integration of VA into CRVS is a complex undertaking. While the potential benefits are great, the success of such a venture relies on a variety of stakeholders, each with their own incentives and impediments to performing their particular role in the process. It is also reliant on the communication mechanisms, IT processes and infrastructure used to facilitate the information flow and eventual use of the COD information for population health statistics. Critical to the sustainability and effectiveness of such a system is the need to introduce changes in a phased manner, with robust monitoring and evaluation processes leading to adaptations and continuous improvements. Usually, that would involve testing during a small-scale pilot and a larger scale implementation before planning for a roll-out across the country. Figure 3 describes an example of a monitoring and evaluation cycle for integrating VA into CRVS.

This figure illustrates a number of stages in the VA cycle, from the effectiveness of data collection (‘are VAs being conducted on all notified deaths in the population or in the designated sample?’) to the publication and dissemination of information arising from VA (‘is the information from VA guiding policy and health programmes?’). It will be important to monitor each of these stages and periodically evaluate, to understand where any problems may be arising. A small number of key indicators will need to be identified at each stage, and these indicators will need to be monitored over time and problem solving employed to understand the root cause of any issues. Critically, strategies to alleviate any identified issues need to be implemented and continuously monitored to ensure that the strategy is working. Some stages relate more to processes (‘is something happening as it should be?’). Others, including data quality assurance, described in more detail in the following section, are more concerned with the quality of the activity (‘is what is happening valid?’). The different stages are also highly dependent on each other. Therefore, a periodic review of the appropriateness of the entire monitoring cycle, with required changes to activities or indicators being monitored, could be performed. This may happen between the pilot and the first phase testing, and between each significant scale-up in the process. The bringing together of all elements of the monitoring and evaluation cycle will be important, given that responsibilities for monitoring are likely to be across a number of different stakeholders. This broader evaluation could be conducted under the auspices of the National Sub-committee on Mortality and Cause of Death and the Task Force on CRVS-VA although other options are possible. VA is a diagnostic tool and therefore its performance will vary based on local epidemiology and the prevalence of specific causes in the mortality cause mix. Locally applicable validation studies could help to determine the external validity of VA results; however, in the absence of locally relevant gold-standard data sets, practical methods for doing this are still in need of development [6,63].

#### Data quality assessment and assurance

Strategies for monitoring data quality will need to be reassessed at appropriate stages in the CRVS VA process maps. It is often unclear in CRVS systems where and when data monitoring and quality assurance occur, as information and data move through the system to become vital statistics. For independence, this may require the engagement of independent support from academia and research. As a CRVS VA system will rely on a strong death notification process and will have implications on the vital statistics reported, the CRVS VA system will need audit trails that allow quality assurance of individual and aggregate data.

After a death has been notified and officially registered, the information should be processed and forwarded to the agency responsible for compiling data (microdata) from the individual records and for analysing the statistics (aggregated data). This is usually the responsibility of the National Statistics Office. Data quality assurance is needed at each stage. At the initial data collection stage, when a death is notified and VA is conducted, the focus of quality assurance is on microdata; that is, the accuracy and completeness of the information contained in each individual record. During the later stages of data compilation, analysis and packaging through to dissemination, reporting and use, quality-assessment processes will primarily focus instead on the accuracy, consistency and plausibility of the aggregated data; that is, the total counts of deaths by age, gender and COD.

A frequently observed problem is the decay of information coverage at various stages along the continuum from the occurrence of an event to its inclusion in the national vital statistics system (Figure 4). Because registration and the production of vital statistics are often the responsibilities of different government agencies, there is a potential for inconsistencies to arise. Any failure to ensure that all registered vital events are reflected in vital statistics is a major concern in terms of the overall quality of CRVS systems.

Different types of errors and inconsistencies can occur between the notification of a death, its official registration and its inclusion in vital statistics. Errors can arise from both under- and over-reporting. Over-reporting can occur when a vital event is noted as a delayed registration without checks being carried out to ensure that it has not already been previously registered in another district. In other cases, re-registration occurs because the initial registration papers cannot be traced, possibly because registration occurred in another area and there is no centralized storage of records, or because of archiving problems. Avoiding duplication of registration requires a well-managed computerized central register and effective archiving, checking and retrieval procedures.

Under-reporting of deaths is far more common than over-reporting. This will be reflected in data showing that the expected annual number of registrations is higher than the figure presented in vital statistics reports. Under-reporting can result because:

- deaths are not notified to the health authorities, burial authorities or other officials, and there is lack of enforcement of public health measures for the safe disposal of bodies
- deaths are notified but not officially registered by the family owing to distance, financial or socio-cultural barriers; in some countries, death registration is not free which, in itself, is a barrier
- there is delayed or incomplete reporting or transfer of the information from civil registration agencies to the vital statistics system
- delayed registrations are treated differently from timely registrations, and not transferred to the vital statistics system
- there is a failure to transmit information on coronial cases or police investigations once they have been resolved and returned to the civil registration system.

Under-reporting can be reduced by introducing an audit system clearly outlining responsibilities for logging all events sent to the vital statistics system, and for recording what is received. Particular challenges arise in relation to the registration of deaths due to accidents or suspicious COD. In most countries, special procedures are in place for registering and certifying accidental or non-natural deaths such as homicides or suicides. Doctors or police called to attend these deaths usually report them to a coroner or judicial authority, and an investigation is carried out to determine the precise COD. In practice, such deaths are frequently under-reported. Stillbirths and neonatal deaths also pose a particular challenge and are chronically under-reported even though they are particularly important outcomes to document. Modern VA questionnaires include the technical possibility of processing information on stillbirths but, in contrast to neonatal deaths, there is no clear guidance about identifying and including them in vital statistics.

## Conclusions

Reliable information on the number of deaths by age, gender and cause is the cornerstone of an effective health information system and national statistics system. However, less than one-third of deaths worldwide are assigned a cause, with the most impoverished nations having the least information while arguably needing it the most. The conventional standard for determining COD is medical certification by a physician familiar with the case and trained in the certification rules and procedures of the ICD. Many deaths in low- and lower-middle-income countries occur in settings without health professionals present or easily accessible to certify the COD. VA is an imperfect tool for ascertaining COD, but the best alternative in the absence of medical certification.

To date, VA has been primarily used in research environments. There are only a few examples where VA has been implemented in a routine manner on a large scale (e.g. Brazil). Recent methodological developments suggest that VA is ready for wider national application in routine CRVS systems. Integrating VA within the CRVS system to capture representative data on a continuous basis is not a trivial undertaking, and there are many unknowns. The successful integration of VA will need to consider the potentially far-reaching system-wide effects in areas such as legal frameworks, medicolegal–ethics issues, human resources, financing, logistics and information systems. Understanding the requirements and potential (positive and negative) system effects of integrating VA is an essential element for an effective implementation. In these early stages and as part of designing any pilot phase, countries need to consider what integrating VA in their CRVS system entails and how the resulting mortality data will be used. Questions about the type of VA method, changes in legislation or regulations, acceptability and feasibility of different approaches and sustainability of the system in the long run could be addressed in early stages of the planning process.

This paper provides an important system-level perspective and overview for how VA can be integrated into routine CRVS systems. It also provides a practical summary checklist of the main points and systems issues to keep in mind when developing the CRVS-VA implementation plan. The checklist identifies one potential set of actions for emerging practices.

## Paper context

More than two-thirds of deaths in low- and lower-middle-income countries occur outside health facilities and are neither registered in national civil registration and vital statistics (CRVS) systems nor assigned a physician-certified COD. The rates and patterns of cause-specific mortality in such countries are changing rapidly owing to epidemiological and health transitions. There is renewed interest globally, regionally and nationally to strengthen CRVS systems to provide such essential evidence to steer national health policies. The means to notify and determine the COD at community level in low-income countries has only been available in research settings capable of implementing population surveillance with verbal autopsy (VA). However, recent developments and innovations in VA methods with the use of tablet computers, mobile communications and diagnostic algorithms obviating the need for physicians to attend every death, open the possibility to integrate VA in routine CRVS systems in such countries. This paper identifies and discusses the systems integration considerations needed to take such decisions and proposes the systemic approaches necessary to conceptualize, design, implement and monitor the introduction of VA in CRVS.
